# Role of Small Molecule Targeted Compounds in Cancer: Progress, Opportunities, and Challenges

**DOI:** 10.3389/fcell.2021.694363

**Published:** 2021-09-08

**Authors:** Guoqiang Sun, Dawei Rong, Zhouxiao Li, Guangshun Sun, Fan Wu, Xiao Li, Hongyong Cao, Ye Cheng, Weiwei Tang, Yangbai Sun

**Affiliations:** ^1^Department of General Surgery, Nanjing First Hospital, Nanjing Medical University, Nanjing, China; ^2^Hepatobiliary/Liver Transplantation Center, The First Affiliated Hospital of Nanjing Medical University, Key Laboratory of Living Donor Transplantation, Chinese Academy of Medical Sciences, Nanjing, China; ^3^Department of Hand Surgery, Plastic Surgery and Aesthetic Surgery, Ludwig-Maximilians University, Munich, Germany; ^4^Department of Musculoskeletal Surgery, Fudan University Shanghai Cancer Center, Shanghai, China

**Keywords:** small molecular compounds, carcinoma, drug resistance, target, therapy

## Abstract

Research on molecular targeted therapy of tumors is booming, and novel targeted therapy drugs are constantly emerging. Small molecule targeted compounds, novel targeted therapy drugs, can be administered orally as tablets among other methods, and do not draw upon genes, causing no immune response. It is easily structurally modified to make it more applicable to clinical needs, and convenient to promote due to low cost. It refers to a hotspot in the research of tumor molecular targeted therapy. In the present study, we review the current Food and Drug Administration (FDA)-approved use of small molecule targeted compounds in tumors, summarize the clinical drug resistance problems and mechanisms facing the use of small molecule targeted compounds, and predict the future directions of the evolving field.

## Introduction

The tumor is a neoplastic proliferation of the abnormal cells of the body formed. Usually, it is abnormal tissue mass on parts of the body. It refers to a new organism generated by abnormal proliferation and the differentiating process of body cells based on a range of initiating and promoting elements. If new organisms develop, they continue to proliferate since the restraints are eliminated. Its growth accepts no normal physiological regulation, while damaging normal tissues and organs. From the perspective of the progression of tumor occurrence and development, the difficulties of the tumor treating process are elucidated below. (1) The etiology of primary tumors remains unclear, and tumors are unlikely to be cured completely. (2) Specific to one group of uncontrolled, infinitely proliferating cells, during their proliferation, the structure and function of normal tissues and organs will be destroyed, and the normal physiological function of the body will be affected. For instance, the immune function of most cases subject to malignant tumors will be reduced to various degrees, causing the body’s immune system to be unable to initiate the regular immune program for the tumor’s inhibiting process. (3) The treating process of the tumor cannot be ended only by the patient’s recent recovery after his treating process, since the tumor is significantly prone to relapse and metastasis, and an active treating process is required during its occurrence. On the whole, the treating process of a malignant tumor consists of the surgical treating process, chemotherapeutic process, radiotherapy, immunotherapy, etc. (Wyld et al., 2015; De Ruysscher et al., 2019; Heinhuis et al., 2019; McLaughlin et al., 2020). The specific treating process plan should be discussed under the diagnosis and treating process mode with the participation of multiple doctors. It should be taken and determined given the nature of the tumor, stage, and the patient’s systemic state.

The new drug developing process shifts its focus to identify molecules and target carcinomas. Carcinoma therapeutic process can be targeted primarily by small molecules and antibodies (Diesendruck and Benhar, 2017; Huck et al., 2018). Antibodies usually exhibit high selective properties. Nevertheless, their aims fall generally into the restriction of the cell surface and are unable to exert drug effect in the membrane. In addition, antibodies should be injected intravenously or subcutaneously for their high molecular weight (e.g., molecule weight of antibody PD-1 Pembrolizumab is 146.286 KD, molecule weight of PD-1 inhibitor BMS-1 is 475.58) and influence of gastrointestinal enzymes on protein digestion. As opposed to those mentioned, small molecule targeted compounds have different selective properties and, due to their small sizes (molecule weight <1000), are capable of binding to various intracellular and extracellular aims orally (Moroz et al., 2016; Wu et al., 2016; Roskoski, 2020). Here, we selected 103 anti-cancer small molecule targeted compounds (Table 1) from the U.S. Food and Drug Administration (FDA) approved drugs to treat cancer^1^. Many of mentioned approvals have shown advantages compared with the cytotoxic chemotherapeutic process, with fewer side influences from the critical treating process inside a recurrence or metastatic environment. Moreover, there are instances regarding small molecule targeted compounds gaining the approval to treat residual disease or as adjuvant therapeutic processes based on therapeutic intent. This review will review the current FDA-approved use of small molecule targeted compounds in tumors, summarize the clinical drug resistance problems and mechanisms facing the use of molecular inhibitors, and predict the future directions of the evolving field.

**TABLE 1:** The application of small molecule targeted compounds in tumors.

**Compound name**	**Indication of tumor type**	**Target**
Erdafitinib	Urothelial carcinoma	FGFR
Everolimus	Breast cancer, Pancreatic cancer, gastrointestinal cancer, gastrointestinal cancer, and lung cancer, Subependymal giant cell astrocytoma, Renal Cell Cancer	mTOR
Abemaciclib	Breast cancer	CDK4/6
Alpelisib	Breast cancer	PI3Kα
Anastrozole	Breast cancer	Aromatase
Exemestane	Breast cancer	Aromatase
Toremifene	Breast cancer	Estrogen receptor
Fulvestrant	Breast cancer	Estrogen receptor
Letrozole	Breast cancer	Estrogen receptor
Palbociclib	Breast cancer	CDK4/6
Ribociclib	Breast cancer	CDK4/6
Lapatinib ditosylate	Breast cancer	EGFR,ErbB2,ErbB4
Olaparib	Breast cancer, ovarian cancer, Ovarian epithelial, fallopian tube, or primary peritoneal cancer, Pancreatic cancer, Prostate cancer	PARP1/2
Megestrol acetate	Breast cancer, Endometrial cancer	progestogen Receptor, Androgen Receptor
Neratinib maleate	Breast cancer	HER2, EGFR
Tamoxifen citrate	Breast cancer, Ductal carcinoma *in situ*	Estrogen receptor
Talazoparib tosylate	Breast cancer	PARP1
Tucatinib	Breast cancer	HER2, ErbB2
Goserelin acetate	Breast cancer, prostate cancer	LHRH agonist
Topotecan Hydrochloride	Cervical cancer, Ovarian cancer, Ovarian cancer	Topoisomerase I
Irinotecan Hydrochloride	Colorectal cancer, Rectal Cancer	Topoisomerase I
Regorafenib	Colorectal cancer, Gastrointestinal stromal tumor, Hepatocellular carcinoma, Rectal Cancer	Ret, Raf-1,VEGFR2
Ziv-Aflibercept	Colorectal cancer, Rectal Cancer	KIT, PDGFRβ, RAF, RET, VEGFR1/2/3
Lenvatinib mesylate	Endometrial carcinoma, Hepatocellular carcinoma, Renal cell carcinoma, Thyroid cancer	VEGFR1/2/3, c-RET
Avapritinib	Gastrointestinal stromal tumor	PDGFRα, c-Kit
Imatinib mesylate	Acute lymphoblastic leukemia, Chronic eosinophilic leukemia or hypereosinophilic syndrome, Chronic myelogenous leukemia, Dermatofibrosarcoma protuberans, Gastrointestinal stromal tumor, Myelodysplastic/myeloproliferative neoplasms, Systemic mastocytosis	v-Abl,c-Kit, PDGFR
Ripretinib	Gastrointestinal stromal tumor	c-Kit,PDGFRα
Sunitinib malate	Gastrointestinal stromal tumor, Pancreatic cancer, Renal cell carcinoma	VEGFR2, PDGFRβ, c-kit
Axitinib	Renal Cell Cancer	VEGFR1/2/3,PDGFRβ,c-Kit
Tivozanib Hydrochloride	Renal Cell Cancer	VEGFR1/2/3
Sorafenib tosylate	Renal Cell Cancer, Hepatocellular carcinoma, Thyroid cancer	Raf-1, VEGFR2, B-Raf
Pazopanib Hydrochloride	Renal Cell Cancer	VEGFR1/2/3
Temsirolimus	Renal Cell Cancer	mTOR
Dasatinib	Acute Lymphoblastic Leukemia (ALL), Chronic myelogenous leukemia (CML)	Abl, Src, c-Kit
Ponatinib Hydrochloride	Acute lymphoblastic leukemia, Chronic myelogenous leukemia (CML)	Abl,PDGFRα,VEGFR2,FGFR1, Src
Enasidenib mesylate	Acute myeloid leukemia (AML)	IDH2
Gilteritinib fumarate	Acute myeloid leukemia (AML)	FLT3/AXL
Glasdegib maleate	Acute myeloid leukemia	Smoothened (Smo)
Ivosidenib	Acute myeloid leukemia (AML)	IDH1
Midostaurin	Acute myeloid leukemia (AML), mast cell leukemia	PKCα/β/γ, Syk, Flk-1
Mitoxantrone Hydrochloride	Acute non-lymphocytic leukemia, Acute non-lymphocytic leukemia, Prostate cancer	Topoisomerase II, PKC
Venetoclax	Acute myeloid leukemia, Chronic lymphocytic leukemia (CLL) or small lymphocytic lymphoma (SLL)	Bcl-2
Acalabrutinib	Chronic lymphocytic leukemia or small lymphocytic lymphoma, Mantle cell lymphoma	BTK
Duvelisib	Chronic lymphocytic leukemia or small lymphocytic lymphoma, Follicular lymphoma	PI3K δ/γ
Ibrutinib	Chronic lymphocytic leukemia and small lymphocytic lymphoma, Mantle cell lymphoma, Mantle cell lymphoma, Waldenström macroglobulinemia	BTK, Bmx, CSK, FGR, BRK
Idelalisib	Chronic lymphocytic leukemia (CLL), Non-Hodgkin lymphoma (NHL), Follicular B-cell non-Hodgkin lymphoma, Small lymphocytic lymphoma	p110δ
Bosutinib	Chronic myelogenous leukemia (CML)	Src/Abl
Nilotinib	Chronic myelogenous leukemia (CML)	BCR-ABL
Cabozantinib-S-Malate	Hepatocellular carcinoma, Medullary thyroid cancer, Renal cell carcinoma	VEGFR2, c-Met, c-RET, c-Kit
Pemigatinib	Cholangiocarcinoma	FGFR
Afatinib dimaleate	Non-small cell lung cancer (NSCLC)	EGFR, HER2
Alectinib	Non-small cell lung cancer	ALK
Brigatinib	Non-small cell lung cancer	ALK,IGF-1R,FLT3,FLT3, EGFR
Capmatinib Hydrochloride	Non-small cell lung cancer	c-Met
Ceritinib	Non-small cell lung cancer	ALK
Crizotinib	Anaplastic large cell lymphoma, Non-small cell lung cancer	ROS1, c-Met, ALK
Dabrafenib mesylate	Anaplastic thyroid cancer, Melanoma, Non-small cell lung cancer	B-Raf
Dacomitinib	Non-small cell lung cancer	EGFR, ErbB2, ErbB4
Entrectinib	Non-small cell lung cancer, Solid tumors	TrkA/B/C,ROS1, ALK
Erlotinib Hydrochloride	Non-small cell lung cancer, Pancreatic cancer	EGFR
Pralsetinib	Medullary thyroid cancer, Non-small cell lung cancer, Thyroid cancer	RET
Gefitinib	Non-small cell lung cancer	EGFR
Lorlatinib	Non-small cell lung cancer	ALK, ROS1
Trametinib dimethyl sulfoxide	Anaplastic thyroid cancer, Melanoma, Non-small cell lung cancer	MEK
Osimertinib mesylate	Non-small cell lung cancer	EGFR
Selpercatinib	Medullary thyroid cancer, Non-small cell lung cancer, Thyroid cancer	c-RET
Tepotinib Hydrochloride	Non-small cell lung cancer	c-Met
Binimetinib	Melanoma	MEK
Encorafenib	Colorectal cancer, Melanoma	RAF
Vemurafenib	Melanoma	B-Raf^V600E^
Plerixafor	Multiple myeloma, Non-Hodgkin lymphoma (NHL)	CXCR4, CXCL12
Panobinostat lactate	Multiple myeloma	HDAC
Selinexor	Diffuse large B-cell lymphoma, Multiple myeloma	CRM1
Fedratinib Hydrochloride	Myelofibrosis	JAK2
Ruxolitinib phosphate	Myelofibrosis, Polycythemia vera	JAK1/2
Copanlisib Hydrochloride)	Follicular lymphoma	PI3Kα/β/γ/δ
Belinostat	Peripheral T-cell lymphoma	HDAC
Zanubrutinib	Mantle cell lymphoma	BTK
Romidepsin	Cutaneous T-cell lymphoma, Peripheral T-cell lymphoma	HDAC1, HDAC2
Tazemetostat Hydrobromide	Epithelioid sarcoma, Follicular lymphoma	EZH2
Umbralisib tosylate	Marginal zone lymphoma, Follicular lymphoma	PI3Kδ
Vorinostat	Cutaneous T-cell lymphoma	HDAC
Rucaparib camsylate	Ovarian epithelial, fallopian tube, or primary peritoneal cancer, Prostate cancer	PARP1
Niraparib tosylate monohydrate	Ovarian epithelial, fallopian tube, or primary peritoneal cancer	PARP1/PARP2
Propranolol hydrochloride	Infantile hemangioma	β-Adrenoceptor
Apalutamide	Prostate cancer	androgen receptor
Bicalutamide	Prostate cancer	Androgen Receptor
Darolutamide	Prostate cancer	androgen receptor
Degarelix	Prostate cancer	GnRH receptor
Leuprolide acetate	Prostate cancer	GnRHR agonist
Enzalutamide	Prostate cancer	androgen-receptor
Flutamide	Prostate cancer	androgen-receptor
Nilutamide	Prostate cancer	androgen-receptor
Relugolix	Prostate cancer	GnRHR
Vismodegib	Basal cell carcinoma	Hedgehog
Sonidegib	Basal cell carcinoma	Smo
Cobimetinib	Melanoma	MEK
Larotrectinib sulfate	Solid tumors	TRK
Imiquimod	Basal cell carcinoma	TLR7
Vandetanib	Medullary thyroid cancer	VEGFR2
Abiraterone acetate	Prostate cancer	CYP17
Tretinoin	Acute promyelocytic leukemia	Retinoic acid receptor, Retinoid X receptor
Sotorasib	Non-small cell lung cancer	K-Ras (G12C)
		

## Classification of Small Molecule Targeted Compounds

The principle of small molecule targeted compounds is to target the molecular biology basis of tumorigenesis, usually to regulate the activity of protein targets. Depending on the type of target, small molecule targeted compounds play different roles. At present, the protein targets of small molecule targeted compounds mainly include enzymes, and receptors. Small molecule targeted compounds can be divided into receptor agonists and receptor antagonists while acting on the receptor.

### Enzyme Inhibitor

Small molecules that act as inhibiting elements of enzymes reduce their catalytic activity by binding to them. Sunitinib and Sorafenib refer to prototypic instances of small molecule multikinase inhibitors (Ito et al., 2019; Procopio et al., 2019; Verschuur et al., 2019; Zahoor et al., 2019; Mammatas et al., 2020; Ruanglertboon et al., 2020). Consistent with most agents pertaining to the mentioned class, these two drugs suppress PDGFR-α, KIT, VEGFR2, and VEGFR1 in various targets. Sunitinib is a multi-target RTK inhibitor, targeting VEGFR2, and PDGFRβ, which also inhibits c-Kit (Mendel et al., 2003). Sunitinib is approved to treat adults with gastrointestinal stromal tumor, pancreatic neuroendocrine tumor and renal cell carcinoma (RCC). A multinational, randomized, double-blind, placebo-controlled phase 3 trial evaluated the clinical effect of Sunitinib in patients with advanced, well-differentiated pancreatic neuroendocrine tumors. The median progression-free survival (mPFS) was 11.4 months in the Sunitinib group versus 5.5 months in the placebo group. The objective response rate was 9.3% of the Sunitinib group and 0% of the placebo group. Clinical trial results show that Sunitinib improves the mPES and objective remission rate (ORR) of patients with pancreatic neuroendocrine tumors. Clinical trial results show that Sunitinib improves the mPES and ORR of patients with pancreatic neuroendocrine tumors. The adverse reactions caused by Sunitinib in the experiment were diarrhea, nausea, vomiting, weakness, and fatigue (Raymond et al., 2011).

Sorafenib is applied in renal cell cancer, hepatocellular carcinoma and thyroid cancer, based on FDA approval. Sorafenib is a multikinase inhibitor of Raf-1, B-Raf, and VEGFR (Wilhelm et al., 2004). In addition to inhibiting the RAF/MEK/ERK signaling pathway, Sorafenib tosylate significantly inhibits the phosphorylation of eIF4E and down-regulates the level of Mcl-1 in liver cancer (HCC) cells in a MEK/ERK-dependent manner, and induces significant apoptosis (Liu et al., 2006). Due to factors such as hepatitis B virus infection, the incidence of liver cancer remains high in the Asia-Pacific region (Petrick et al., 2016). The application of Sorafenib in liver cancer is prevalent. Therefore, the clinical effect of Sorafenib in patients in the Asia-Pacific region is an important issue. A phase III randomized, double-blind, placebo-controlled trial in patients of the Asia-Pacific region with advanced hepatocellular carcinoma evaluated the efficacy and safety of Sorafenib. A total of 271 patients from China, South Korea, and Taiwan was enrolled in the study. Finally, the median overall survival (MOS) of the sorafenib treatment group was 6.5 months, and that of the placebo group was 4.2 months. The median time to progression was 2.8 months in the Sorafenib group and 1.4 months in the placebo group. The most common grade 3/4 drug-related adverse events were hand-foot skin reactions (10.7%), diarrhea (6.0%), and fatigue (3.4%), and these adverse events rarely lead to discontinuation (Cheng et al., 2009). Dose-restricting toxic influences are consistent in such a drug class and receive the predominant motivation through the VEGFR inhibiting process. One subset of carcinomas is significantly addicted to one oncogene or certain molecular defects capable of exploiting channels selectively (e.g., those impacting DNA repairing or apoptotic process). Selection-related small molecule targeted compounds are capable of effectively antagonizing the expected aim and eliminating the off-aim inhibiting process, probably causing dose decrease or reduction of intolerable side-influences. EGFR inhibitors (e.g., Gefitinib and Erlotinib) received the initial development with no single case selecting method and exhibited appropriate efficacy in cases subjected to non-small-cell lung carcinoma undergoing the pretreating process using the normal cytotoxic chemotherapeutic process (Shepherd et al., 2005; Kim et al., 2008).

### Receptor Antagonist

In normal scenarios, the receptor is activated after binding to its ligand (signal), and the signal is transmitted down, producing certain biological phenomena. Some compounds can bind to the receptor, act in the identical role as the ligand and activate the receptor. Such compounds are receptor agonists. In the presence of an agonist, an antagonist antagonizes the agonistic effect of the agonist on the receptor, and the antagonist alone exerts no effect on the receptor (Pease, 2017; Arena et al., 2018). Prostate carcinoma is the most common carcinoma among aged males in western countries and more aggressive and lethal castration-resistant prostate carcinoma (CRPC) often occurs after the androgen deprivation therapeutic process (ADT). The high expression of androgens and androgen receptors (AR) is closely related to prostate carcinoma. Efficient AR antagonists, such as Enzalutamide and ARN-509, could be employed as potent anti-prostate carcinoma agents (Ge et al., 2018). Selinexor, combined with Dexamethasone, has gained approval for patients with Penta-refractory multiple myeloma (MM) by FDA. As we all know, transcription takes place in the nucleus, separated from the translation of cytoplasm. Nuclear pore complex (NPC) assures adequate cell function of transmission of genetic information, acting as a connection bridge. Large molecule (40 kDa) cargo requires a specific transport receptor protein to pass through NPC, such as chromosome region maintenance-1 (CRM-1) which is the target site to selinexor. Selinexor is a slow reversible, oral and selective Inhibitor of Nuclear Export compound, binding covalently to cysteine 528 in the cargo-binding nuclear export slot of CRM-1 and forcing the nuclear to retain and activate tsp, GR and IkBa; as well as a nuclear-cytoplasmic export limitation and mRNA translation of eIF4E-bound oncoprotein (Sun et al., 2013; Neggers et al., 2015). Consequently, Selinexor triggers cell cycle arrest and apoptosis in the solid and hematologic tumor cells. The part II of phase 2b Selinexor Treatment of Refractory Myeloma (STORM) trial (NCT02336815, part II) enrolled 122 heavily pretreated patients with TCR/Penta- exposed MM. ORR was observed in 26.2% of patients; ORR was observed in 25.3% of the five refractory patients. The clinical benefit rate (CBR) was 39%, the median remission period was 4.4 months, and the most prolonged remission periods exceeded 18 months. mPFS was 3.7 months for all patients, and 2.1 months for non-responders. MOS was 8.6 months for all patients, and 1.9 months for non-responders (Chari et al., 2019; Podar et al., 2020). Lenvatinib inhibits vascular endothelial growth factor receptor 1–3, fibroblast growth factor receptor 1–4, platelet-derived growth factor receptorα, and KIT, approved for Endometrial carcinoma, hepatocellular carcinoma, RCC, thyroid cancer (Matsui et al., 2008). In hepatocellular carcinoma, it is used as a first-line treatment for patients whose disease cannot be removed by surgery. Kudo et al. (2018) concluded that the overall survival rate of Lenvatinib is not inferior to Sorafenib in untreated advanced hepatocellular carcinoma through an open, phase 3, multi-center, non-inferior quality trial. In the study, 954 qualified patients were randomly assigned to Lenvatinib group (*n* = 478) or sorafenib group (*n* = 476). The median survival time of Lenvatinib is 13.6 months and not less than the median survival time of Sorafenib 12.3 months, which meets the criteria of non-inferiority. The most common adverse events of any grade in the Lenvatinib group were hypertension [201 cases (42%)], diarrhea [184 cases (39%)], loss of appetite [162 cases (34%)], weight loss [147 cases (31%)]. Palmar and plantar red sensory disturbances [249 cases (52%)], diarrhea [220 cases (46%)], hypertension [144 cases (30%)], and decreased appetite [127 cases (27%)] in the Sorafenib group. These results show that Lenvatinib has a better clinical benefit than Sorafenib (Al-Salama et al., 2019).

### Receptor Agonist

Although enzyme inhibitors and receptor antagonists show superior clinical efficacy of various cancers in small molecule targeted compounds, receptor agonists exert a specific effect in cancer endocrine therapy. Androgen deprivation therapy is an essential treatment of advanced and metastatic prostate cancer, including surgical castration and medical castration (Singer et al., 2008) [e.g., Bicalutamide and Nilutamide, Ketoconazole, or Corticosteroids (Teo et al., 2019)], and its purpose is to remove the role of nutrition androgen for prostate cancer. Luteinizing hormone-releasing hormone (LHRH) agonists are a type of androgen deprivation therapy. Goserelin acetate and Leuprolide acetate are LHRH agonists, continuous application can down-regulate the LHRH receptor and cause desensitization of the gonadotropin cell receptor after a short period of high stimulation, resulting in the obstruction of the secretion of testosterone in the testicular stromal cells (Stojilkovic et al., 1994; Shim et al., 2019). Compared with LHRH agonists, anti-androgen drugs show poor survival results, so LHRH agonists have a more comprehensive range of applications in the treatment of prostate cancer (Seidenfeld et al., 2000). A retrospective study showed that, unlike single antiandrogen therapy, LHRH agonist monotherapy provides long-term and durable control of localized prostate cancer. For those anti-androgen monotherapy ineffective patients, LHRH agonist is also an effective treatment option (Raina et al., 2007). In addition to prostate cancer, LHRH agonist Goserelin Acetate is also approved by FDA for the treatment of breast cancer.

## The Application of Small Molecule Targeted Compounds in Cancers

In this review, we classify the existing small molecule targeted compounds for the carcinoma therapeutic process into the following four categories according to the different channels targeted. We have screened out 103 small molecule targeted compounds for cancer treatment from the drugs approved by the FDA. Specific targets and detailed information are shown in Table 1.

### Targeting DNA Damage/Repair Channel

**FIGURE 1:**
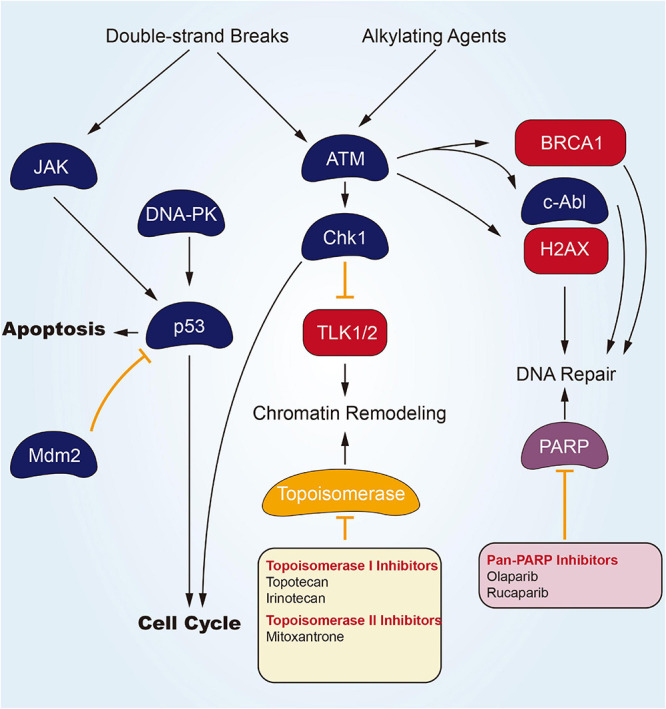
Small molecule targeted compounds targeting DNA damage/repair channel in cancers.

DNA undergoes continuous exposure to various damages of endogenous and exogenous elements. Their repairing process is critical to maintain genomic integrity for a long, safe life. An incomplete or unsuccessful DNA repairing process causes genomic instability and cellular transforming process. The DNA damage response (DDR) is a complex cellular network activated by DNA damage with the final aim to repair the DNA damage and restore genomic integrity, through the involvement of many intracellular channels (Ciccia and Elledge, 2010; O’Connor, 2015). DDR is vital for maintaining genomic integrity, as suggested by the fact that germline mutations in DDR genes are associated with an increased risk of tumors (Klinakis et al., 2020). Given its effect on DDR and DNA repairing, DNA-dependent protein kinase (DNA-PK) inhibitors are expedited to be developed. According to Figure 1, several potential modes of small molecular parts pertaining to DNA helicases are presented. Olaparib refers to one potent poly adenosine diphosphate ribose polymerase (PARP) inhibitor, inducing synthetic lethality in BRCA1/2-lacking tumor cells. In December 2014, Olaparib gained approval from the European Medicines Agency for its application in the therapeutic maintenance process of cases under the effect of BRCA1/2-mutated ovarian carcinomas (OC) in respondence to platinum-related chemotherapeutic process. Moreover, in the US, Olaparib gained expedited approval from the US FDA as a monotherapy agent for cases subjected to deleterious or suspected deleterious germline BRCA-mutated advanced OC and those administrated using three or more prior lines of chemotherapeutic process (Musella et al., 2015). Moore et al. (2018) have completed a phase III trial that was random and, double-blind to assess the Olaparib efficacy in patients of newly diagnosed advanced ovarian cancer primary peritoneal cancer, or fallopian tube cancer (or a combination thereof) with a mutation in BRCA1, BRCA2, or both (BRCA1/2). Of the 391 randomized patients, 260 were assigned to receive Olaparib treatment and 131 accepted placebo treatment. A total of 388 patients have concentratedly confirmed germline BRCA1/2 mutations, and two patients have concentratedly confirmed systemic BRCA1/2 mutations. After a median follow-up of 41 months, disease progression or death risk Olaparib lower than placebo 70% (Kaplan Meier estimated 3-year probability of freedom from disease progression and death was 60% vs. 27%). Specific to phase II clinical trials, covering cases subjected to advanced CRPC, Olaparib seems to be efficacious and well-tolerated (De Felice et al., 2017). And de Bono et al. (2020) finished a randomized, open-label, phase III trial, evaluating Olaparib in metastatic CRPC patients who had disease progression after receiving a novel hormonal drug. In cohort A of at least one alteration in BRCA1, BRCA2, or ATM, the imaging-based progression-free survival of the Olaparib group was significantly longer than that of the control group (median, 7.4 vs. 3.6 months), objective response rate and the time to pain progression were confirmed to obtained a significant benefit. In cohort A, the MOS of the Olaparib group and the control group were 18.5 and 15.1 months, respectively, and 81% of progressing patients switched to Olaparib treatment. The overall population of cohort A and cohort B acquired an apparent benefit of Olaparib for imaging-based progression-free survival.

Inhibitors of topoisomerase I (Top1) that result in stalled Top1 cleavage complexes (Top1cc) are commonly employed against carcinoma. Combination chemotherapeutic process with DNA repair inhibitors can potentially improve response to the mentioned widely used chemotherapeutics. One line of inquiry focuses on inhibitors of tyrosyl-DNA phosphodiesterase 1 (Tdp1), a repair enzyme for Top1cc. Tdp1 catalyzes the hydrolysis of DNA adducts covalently linked to the 3’-phosphate of DNA, including Top1-derived peptides and 3’-phosphoglycolates. Tdp1 inhibitors (Topotecan Hydrochloride Irinotecan Hydrochloride) should synergize not only with Top1-targeting drugs (Camptothecins, Indenoisoquinolines), but also with bleomycin, topoisomerase II (Top2) inhibitors (Etoposide, Doxorubicin) and DNA alkylating agents (Huang et al., 2011).

### Targeting Endocrinology and Hormones Channel

**FIGURE 2:**
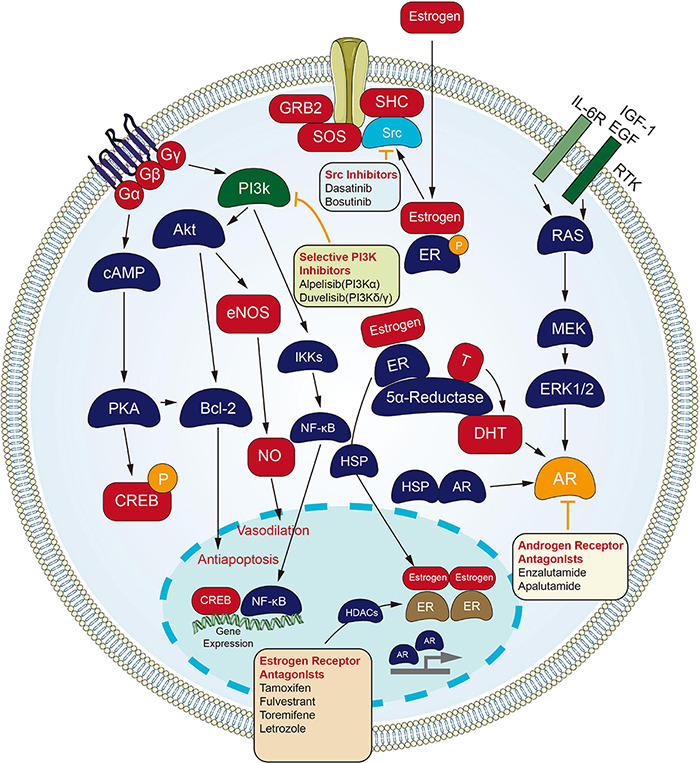
Small molecule targeted compounds targeting endocrinology and hormones channel in cancers.

Inhibitors targeting estrogen and AR are of great significance for the treating process of endocrine tumors (Figure 2). Fulvestrant, a selective estrogen receptor down-regulator (SERD), which blocks the proliferation of breast carcinoma cells (Wakeling et al., 1991), is an effective endocrine treating process for women with hormone-sensitive advanced breast carcinoma. Fulvestrant is approved as a first-line treating process for metastatic breast carcinoma. Still, its use in this setting may be limited to situations where the combination with CDK4/6 inhibitors is not available. Response to Fulvestrant was particularly durable in cases subjected to bone-only metastatic disease (Soleja et al., 2019). The combination of Fulvestrant with CDK4/6 inhibitors has shown superior efficacy compared to the monotherapy process in cases subjected to metastatic hormone receptor-positive breast carcinoma who have progressed or relapsed on prior Tamoxifen or Aromatase inhibitor therapeutic process (Sledge et al., 2017). On the whole, resistance to hormonal therapy displays an association with PIK3CA and ESR1 mutations. Alternative hormone resistance channels may be mediated by upregulation of PI3K/AKT, HER2, FGFR, and IGF channels (Beeram et al., 2007; Zhang et al., 2011). Fulvestrant regimens in combination with emerging targeted agents are being developed to overcome endocrine-resistant breast carcinoma. Most recently, Fulvestrant with Alpelisib, an α-selective PI3K inhibitor, has been recently approved for cases who have progressed on an endocrine therapeutic process with PIK3CA mutated breast carcinoma (Soleja et al., 2019). The FDA approval is based on a randomized, double-blind, placebo-controlled SOLAR-1 study of Alpelisib plus Fulvestrant and placebo plus Fulvestrant. The median PFS of the Alperizil combined with Fulvestrant group was 11.0 months, while the median PFS of the placebo plus Fulvestrant group was 5.7 months. The MOS of the Alpelisib plus Fulvestrant group has not been reached, while the MOS of the Fulvestrant control group is 26.9 months. The most common adverse reactions are increased blood sugar, increased creatinine, decreased lymphocyte count, increased gamma-glutamyl transferase and other laboratory abnormalities (Narayan et al., 2021).

Androgen receptor signaling refers to one critical channel in prostate carcinoma, and cases receive the initial administration with ADT. Cases that stopped responding to ADT process were recognized as having CRPC, which is still dependent on AR signaling. Enzalutamide, an orally available AR inhibitor, was initially approved by the US FDA for the treating process of cases subjected to metastatic CRPC that have previously received docetaxel. The indication was subsequently extended to include all cases subjected to metastatic CRPC, and most recently to include cases subjected to non-metastatic CRPC (Sternberg, 2019).

### Targeting Metabolism Channel

**FIGURE 3:**
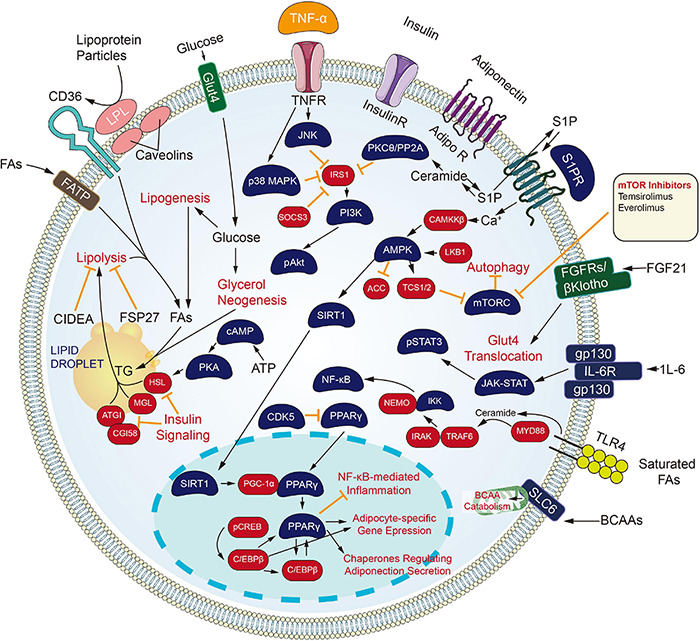
Small molecule targeted compounds targeting metabolism channel in cancers.

Inhibiting the process of metabolic channels also brings a new dawn to the carcinoma treating process (Figure 3). Cytochrome CYP450 cyclooxygenase catalyzes the epoxidation of polyunsaturated fatty acids, including arachidonic acid, eicosapentaenoic acid, and docosahexaenoic acid. The product of arachidonic acid refers to one feasible lipid facilitating angiogenesis and facilitates tumors developing and growing processes. Besides, derivatives of eicosapentaenoic acid and docosahexaenoic acid limit angiogenesis and have protecting effects under certain pathological conditions covering carcinoma (Sausville et al., 2019; Guengerich, 2020). Inhibitors against the CYP450 family came into being. Since androgen signaling critically impacts the proliferating and metastatic processes of prostate carcinoma, ADT or castration therapeutic process is recognized as the backbone of the treatment process for newly diagnosed metastatic prostate carcinoma. Nevertheless, all men experience disease progression on ADT to a state known as metastatic castration-resistant prostate carcinoma (mCRPC), as driven by AR signaling or intratumoral androgen synthesis continuously. For this reason, the extragonadal ablation of androgen synthesis from pregnane precursors holds much promise. One inhibitor of cytochrome P450 17α-hydroxy/17,20-lyase (CYP17) enzymes, Abiraterone Acetate, has already been approved for men with mCRPC. Newer CYP17 inhibitors continue to be developed, which are either more selective or have concomitant inhibiting actions on AR signaling. The mentioned include VT-464, Orteronel, and Galeterone (Alex et al., 2016).

Retinoids and their naturally metabolized and synthetic products [All-trans retinoic acid (ATRA), 13-cis retinoic acid and Bexarotene] induce differentiating processes in various cell types. Retinoids exert their actions primarily through binding to the nuclear retinoic acid receptors (α, β, γ), which are transcriptional and homeostatic regulators with functions that are often compromised early in the neoplastic transforming process (Alvarez et al., 2007).

All-trans retinoic acid combined with chemotherapy is the standard treatment for acute promyelocytic leukemia (APL), with a cure rate of more than 80% (Lo-Coco et al., 2013). Tretinoin is approved by FDA to be used with arsenic trioxide to treat APL in patients whose cancer has a certain type of chromosome mutation that affects the PML gene and RARA gene.

Besides, the retinoids have been investigated broadly for carcinoma prevention and treatment, primarily attributed to their capacity for inducing cellular differentiating process and arrest proliferating process. RA-regulated tumor suppressor genes, when expressed, can inhibit tumor growth (Houle et al., 1993). Among the three RARs, RARβ has been well known for its tumor suppressive effects in epithelial cells (Tang and Gudas, 2011). It is also becoming increasingly clear that RARβ expression is lost early in carcinogenesis or is epigenetically silenced in many solid tumors (Sirchia et al., 2000), providing an opportunity for emerging treating strategic processes to be investigated with the use of retinoids as well as epigenetically-related modifying elements promoting silenced genes to be re-expressed.

As revealed from Ai et al. (2018) the proliferating and migrating processes were inhibited and the apoptosis of Non-small cell lung cancer (NSCLC) cells was accelerated by Bexarotene (Retinoic acid). Moreover, overexpressed slc10a2 in NSCLC cells can more significantly limit the proliferating and migrating processes, and promote apoptosis under the treating process of bexarotene. As opposed to those mentioned, the opposite results were obtained after the slc10a2 gene was silenced in NSCLC cells administrated with Bexarotene. Furthermore, TSC2, LKB1, P53, P21, PTEN, caspase 7, caspase 3 expressing states were increased and the expression of Bcl-2, cyclin D1, c-FLIP were declined in NSCLC cells and slc10a2 overexpressed NSCLC cells with the treating process of Bexarotene. The opposite situations were seen after the slc10a2 gene was silenced in NSCLC cells. Further studies revealed the increased expression of slc10a2 activated the expression of peroxisome. The mentioned results suggest that Bexarotene inhibits the viability of lung carcinoma cells via slc10a2/PPARγ/PTEN/mTOR signaling channel (Sirchia et al., 2000).

### Targeted Angiogenesis Channel

**FIGURE 4:**
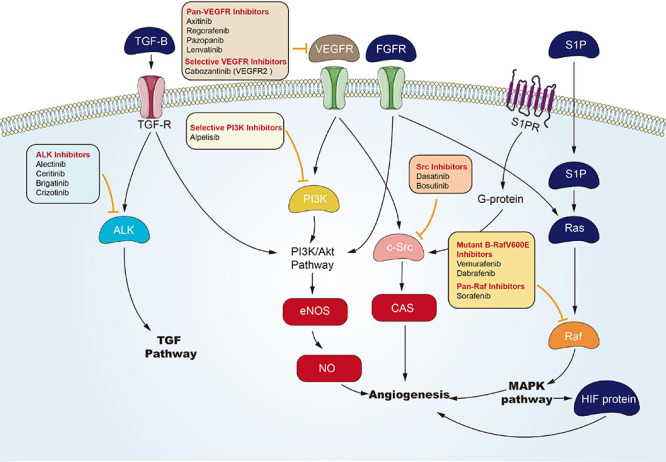
Small molecule targeted compounds targeting angiogenesis channel in cancers.

Angiogenesis is considered the forming process of novel blood vessels from vessels that exist in advance. It refers to one complicated multiple-step process, showing tight regulation under a delicate balance between inducers and inhibitors that act together to maintain physiological homeostasis (Hanahan and Folkman, 1996). Nevertheless, tumor under proliferation will activate angiogenesis through the shift of the balance of inducers and inhibitors to one pro-angiogenic result, to fulfill its increased demand of oxygen and nutrients (Carmeliet, 2005). Environmental hypoxia in tumors appears as one primary element that turns on an ‘angiogenic switch’ by enhancing expression and activation of transcription element hypoxia-inducible-element-1 (HIF-1) channel or HIF-1-independent channels, and it induces the expression of multiple genes contributing to the angiogenic process (Pugh and Ratcliffe, 2003). In Folkman (1972) proposed anti-angiogenesis as a new anticarcinomaous strategy initially. Seventeen years later, vascular endothelial growth element A’s (VEGFA) isolation and cloning became one landmark to clarify angiogenic mechanism (Keck et al., 1989), and underpinned the emerging field of research into anti-angiogenic treating processes for carcinoma. The active relevant studies resulted in FDA approval of Bevacizumab (a monoclonal antibody for VEGFA) as the initial anti-angiogenic drug for colorectal carcinoma in 2004 (Hurwitz et al., 2004). Over the last decade, numerous potential anti-angiogenic targets have been identified in sequence, covering cell adhesion molecule, tumor-associated stromal cell, matrix metalloproteinase, and fibroblast growth element. To be specific, VEGFs and their receptors (VEGF receptor-1, VEGF receptor-2, and VEGF receptor-3), exhibiting tyrosine kinase activity, play critical roles in angiogenesis (Ferrara et al., 2003). Therefore, most of the angiogenesis inhibitors are developed targeting VEGFs or their receptors. To date, a large number of angiogenesis inhibitors have been discovered and developed, ranging from monoclonal antibodies, endogenous angiogenesis peptide inhibitors, to small molecule drugs (Figure 4). In the past few years, many emerging small molecule inhibitors have been synthesized, covering phosphatidylinositol 3-kinase, benzoxazines targeting receptor tyrosine kinase, 6-(pyrimidine-r-acyloxy)-naphthalene-1-carboxamides, and indoline-2-one group and noreremophilane-based inhibitors, respectively (Al-Rawi et al., 2015; Muthukumarasamy et al., 2016). The mentioned emerging series of compounds showed potential for treating solid tumors in pre-clinical experiments. Furthermore, natural products from marine, bacteria, or herb received the development and demonstration of anti-angiogenic properties (Wang and Miao, 2013; Ehlers et al., 2016). Advanced RCC is usually treated with targeted drugs that block blood vessels. Sorafenib and Axitinib are two commonly used drugs. The mechanism of action of Sorafenib has been described above. Axitinib is an inhibitor of VEGFR-1, VEGFR-2, VEGFR-3, PDGFR-β, and c-KIT. Axitinib can block the autophosphorylation of VEGFR, endothelial cell viability regulated by VEGF, microtubule formation, and downstream signals (Rössler et al., 2011). There are related studies on the comparison of the effects and safety of these two drugs. Brian I Rini et al. (2011) conducted a randomized phase 3 study to compare the clinical efficacy and safety of Axitinib and Sorafenib as a second-line treatment for patients with metastatic RCC. Among the 723 patients enrolled, they were randomly divided into the Axitinib group (361 cases) and the Sorafenib group (362 cases). The result was that the mPES with Axitinib was 6.7 months, and the mPES with Sorafenib was 4.7 months. In terms of safety, 14 of 359 patients (4%) who received Axitinib and 29 (8%) of 355 patients who received Sorafenib stopped treatment due to side effects. The most common adverse events were diarrhea, hypertension, and fatigue of Axitinib group, and diarrhoea, palmar-plantar erythrodysesthesia, and alopecia of sorafenib group. Therefore, Axinitinib shows better clinical benefit in the treatment of advanced renal cancer compared with Sorafenib.

### Targeted Immune Checkpoint Blockers

Since the FDA approved Ipilimumab which is a human CTLA-4 blocking antibody and alters the adaptive immune system (Carreau and Pavlick, 2019) for the treatment of melanoma in 2011, cancer immunotherapy has become an epoch-making achievement and has achieved some exciting clinical applications. Tumor evolution is to avoid immune attack. The tumor microenvironment is immunosuppressive and can be suppressed by immune checkpoints (Baumeister et al., 2016; Hoos, 2016). Immunotherapy aims at reactivating repressive immune cells of cancer patients and acquires exciting results, especially for immune checkpoint blockers of immune monoclonal antibodies, such as PD-1 antibody, PD-L1 antibody, as well as CTLA-4 antibody (Carbone et al., 2017). The most significant benefit of immune checkpoint inhibitor therapy is to restore the function of the immune system, use immune function to clear the specificity of the tumor, and form a lasting memory similar to the antigen. In addition to mentioned agents, molecule inhibitors of immunity are gradually becoming the focus of immunotherapy due to the advantages that they can be absorbed orally, have a small molecular weight and can penetrate cell membranes to act in cells. PD-1, PD-L1, and CTLA-4 are the three most popular immune targets. PD-1 is a member of the CD28 family and is an inhibitory receptor expressed on activated T cells, B cells, macrophages, regulatory T cells (Tregs) and natural killer (NK) cells. It has two binding ligands PD-L1 and PD-L2 (B7 family) expressed on normal cells. The combination of PD-1 with either ligand can inhibit T cell activity, induce T cell tolerance, and inhibit Proliferate, reduce the immune response of T cells and induce cell death, thereby preventing immune cell activation and killing normal cells, and inhibiting the proliferation, differentiation and secretion of antibodies of B cells. CTLA-4 has the same ligand as CD28, but CTLA-4 transmits inhibitory signals. The affinity of CTLA-4 to the ligand is significantly higher than CD28 (Greaves and Gribben, 2013; Kuzume et al., 2020). Some tumor cells will use this mechanism to secrete a large amount of PD-L1/L2 to reduce T cell activation and antigen-specific T cell response, result in evading immune surveillance (Zou et al., 2016). Although no related small molecule targeted compounds have been approved for marketing, some drugs have shown certain therapeutic prospects. In 2016, CA170 was the first to obtain a new drug research application for small molecule immune checkpoint inhibitors. CA-170 is the only small molecule modulator that can be taken orally for PD-L1 and VISTA proteins in clinical trials, and is an essential immune activation negative checkpoint modulator. According to reports, the treatment results are similar to FDA-approved monoclonal antibodies to a specific extent, and overcome the latter’s high production cost and side effects (Musielak et al., 2019). In 2016, CA-170 became the first small molecule immunotherapy to enter the Phase I clinical trial of advanced solid tumors and lymphomas (Shaabani et al., 2018). Stimulator of interferon genes (STING) is an immunostimulatory small molecule target, mainly distributed in immune-related tissue cells, such as thymus, spleen, and peripheral blood leukocytes. STING activation leads to nuclear translocation of transcription factors, induces the expression of interferon (INF) and cytokines, promotes the aggregation and activation of T cells, and then kills tumor cells. Zaidi et al. (2021) evaluated ADU-S100 anticarcinoma effect on esophageal adenocarcinoma, which is a STING agonist. The tumor volume in the ADU-S100 and ADU-S100 plus irradiation groups decreased by 30.1 and 50.8%, respectively, and the tumor volume in the placebo group and placebo plus irradiation group increased by 76.7 and 152.4%, respectively. INFβ tumor necrosis factor-α IL-6 and CCL-2 were significantly upregulated in the treatment group compared with placebo. Currently, ADU-S100 is being evaluated in clinical trials (NCT03172936) (Zaidi et al., 2021).

### Others

In recent years, small molecule targeted compounds have targeted drug-resistant mutation sites to solve the drug-resistant problem of targeted drugs. NSCLC is the focus of targeted therapy with small molecule inhibitors, but the problem of drug resistance is a complex problem to solve. KRAS p.G12C mutation occurs in 13% of NSCLC and 1 to 3% colorectal cancer and other cancers. Sotorasib is a selective and irreversible KRASG12C targeted small molecule, and has exciting effects. Hong et al. (2020) conducted a phase 1 trial of Sotorasib in patients of advanced solid tumors with KRAS p.G12C mutations. In the subgroup of patients with NSCLC, 32.2% (19 patients) had a clear objective response (complete or partial response), and 88.1% (52 patients) had disease control (objective response or stable disease); The mPES time was 6.3 months. In the colorectal cancer subgroup, 7.1% (3 patients) received a confirmed response, 73.8% (31 patients) received disease control, and the mPES was 4.0 months. Reactions have also been observed in patients with pancreatic cancer, endometrial cancer, appendix cancer, and melanoma. And in the CodeBreak 100 phase II trial, Sotorasib elicited a response in more than one-third of patients and resulted in a mPES of nearly 7 months. Amgen has submitted an application for approval of the drug to the FDA and the European Medicines Agency (No author list, 2021). On May 29, 2021, the FDA announced the accelerated approval of Sotorasib (Lumakras) for the treatment of NSCLC patients with KRAS G12C mutations. These patients receive at least one pre-systemic treatment. Sotorasib is the world’s first anti-tumor drug targeting mutant KRAS protein. T790M mutation and MET mutation are the secondary drug resistance mutations of EGFR in lung cancer patients after receiving EGFR inhibitor treatment (Suda et al., 2017). Small molecule targeting compounds that target T790M, such as Osimertinib, have been approved for marketing and have achieved better efficacy than chemotherapeutic drugs (Mok et al., 2017). Until the revolutionary anti-cancer drug Tepotinib was approved for the market, the gap that there was no small molecule drug for MET mutations was broken. In an open-label phase 2 study, Tepotinib was used in advanced NSCLC patients with skipping mutations in exon 14 of MET, and about half of the patients had an objective response. Peripheral edema was the main toxic reaction of grade 3 and above (Paik et al., 2020). The FDA has approved Tivozanib of VEGFR inhibitor for the treatment of adult patients with relapsed or refractory advanced RCC who have received two or more systemic therapies. Tivozanib has become the first therapy approved for this indication. ATP-binding cassette (ABC) transporter can mediate multidrug resistance of tumor cells. Yang et al. (2014) reported that Tivozanib had the activity of reversing multidrug resistance mediated by ABCB1 and ABCG2 transporters. Therefore, Tivozanib has revolutionized the treatment of kidney cancer. A phase 3, multicenter, randomized, controlled, open-label study compared Tivozanib and Sorafenib in the treatment of advanced renal cell carcinoma (TiVo-3). The results of the study show that Tivozanib as a third-line or fourth-line treatment can improve the progression-free survival rate of patients with metastatic kidney cancer, and it is better tolerated than Sorafenib (Rini et al., 2020).

## Mechanism of Resistance of Small Molecule Inhibitors

Drug resistance is a common phenomenon in the treatment of cancer, which is divided into acquired resistance and natural resistance. In cancer chemotherapy, many cancer patients begin to be sensitive to chemotherapeutic drugs. As the treatment progresses, cancer cells will develop resistance through some mechanisms, leading to treatment failure, and the same phenomenon will occur with small molecule inhibitors. Drug resistance includes drug inactivation, drug target alteration, drug efflux, DNA damage repair and epithelial-mesenchymal transition (EMT). The development of drug resistance in the treatment of acute myeloid leukemia with cytarabine is an example of drug inactivation. Cytarabine is activated by multiple phosphorylation, mutation or down-regulation of the phosphorylation pathway will inactivate cytarabine and cause drug resistance (Sampath et al., 2006). Small molecule inhibitors for the treatment of signal kinases will cause changes in drug targets, for example, certain lung cancer patients with EGFR mutations developed resistance to EGFR inhibitors within one year, and EGFR-T790M gatekeeper mutation was reported in half of the cases (Bell et al., 2005). Drug efflux is a most interesting mechanism for researchers to study, involving the enhanced efflux to reduce drug content. ABC transporter is not only a normal physiological phenomenon, but also a mechanism of cancer cell resistance. Multidrug resistance protein 1 (MDR1) is a transporter with extensive substrate specificity and the ability to excrete many exogenous substances from the cell, including Vinblastine, podophyllotoxin, Anthracycline, Taxanes, and kinase inhibitors (Gottesman et al., 2002; Szakács et al., 2004). Hence, MDR1 protects cancer cells from drugs.

DNA damage response plays a vital role in resisting drugs that induce DNA damage to kill cancer cells. Though PARP inhibitors target DDR to prevent DNA repair and display a predominant effect with chemotherapy, other DDR mechanisms can be adjusted up to compensate for dysfunctional pathways (Housman et al., 2014). In addition to EMT being an essential mechanism of solid tumor metastasis, EMT is also the cause of drug resistance. It is reported that in head and neck squamous cell carcinoma (HNSCC) cells, the NGF/TrkA axis confers resistance to the EGFR inhibitor Erlotinib through the EMT process. *In vitro* and *in vivo* models, blocking TrkA signaling significantly reverses EMT and makes HNSCC cells sensitive to Erlotinib (Lin et al., 2020). There are still many questions about the drug resistance mechanism of inhibitors, which urgently need to be studied and elucidated, so that the clinical application of the drug can be better.

## The Combined Therapy of Small Molecule Targeted Compounds

The purpose of combination therapy is to overcome and reverse the drug resistance of inhibitors, reduce side effects and achieve better therapeutic results through the combination of multiple pathway inhibitors. Single-use of small molecule inhibitors to treat cancer often has mutation sites that affect drug treatment. Although few inhibitor combinations are approved by FDA, the combination of inhibitors can solve this problem. MEK inhibitor AZD6244 inhibits the activation of ERK of colon cancer cells, while AZD6244 promotes JAK2-STAT3 signaling activation which induces colon cancer cell resistance to inhibitor. Jin et al. (2019) have shown that the combined effect of MEK inhibitor AZD6244 and JAK2-STA T3 inhibitor AG490 can significantly inhibit cell viability, induce cell apoptosis, and ultimately inhibit the activation of ERK and JAK2-STAT3 signals. The combination of AZD6244 and AG490 has shown better effects than single drugs *in vivo* and *in vitro*. Bruton’s tyrosine kinase (BTK) inhibitor Ibrutinib and Acalabrutinib appear to be highly effective. However, BTK inhibition enhances the reliance of mitochondria on BCL-2 without significantly changing the overall activation of mitochondria. The selective BCL-2 inhibitor Venotclax treatment improves the overall mitochondrial activation without increasing dependence on BCL-2. Therefore, BCL-2 inhibitor and BTK inhibitor have synergistic and complementary therapeutic effects (Deng et al., 2017). Toxicity and side effects, including fatigue, skin, mucous membrane, and gastrointestinal adverse reactions, limit the use of different drug dosages and treatment schedules. In some combinations of inhibitors, the combination group shows lower side effects and better therapeutic effects than the single-use group. The phase III clinical trial of patients with metastatic melanoma with BRAF V600E or V600K mutations completed by Robert et al. (2015) confirmed that the combination of the BRAF inhibitor Dabrafenib/Vemurafenib and the MEK inhibitor Trametinib has a better therapeutic effect and Unelevated overall toxicity. The 12-month overall survival rate in the combined treatment group was 72%, and the overall survival rate in the Vemurafenib group was 65%. The mPES was 11.4 months in the combined treatment group, and 7.3 months in the Vemurafenib group. The objective effective rate was 64% in the combined group and 51% in the Vemurafenib group. The rates of serious adverse events and drug discontinuation were similar in the two groups. Cutaneous squamous cell carcinoma and keratoacanthomas were 1% and 18% in the combination treatment group and Vemurafenib group, respectively.

## Conclusion and Perspectives

Small molecule targeted compounds have been developed in clinical medicine for decades, prolonging the survival time of cases subjected to advanced or refractory tumors. However, there are still insufficient approved drugs for practical use for several reasons. Even if a variety of enzymes are identified, inhibitors only target specific kinase targets for EGFRs, FGFRs, VEGFRs, JAK, PI3K, and CDK and other new drugs, resulting in most of the drugs studied having similar structures and mechanisms compared with the inhibitors used. Our detailed use of new gene-editing techniques to construct more comprehensive and effective protein kinase gene knockout libraries and to screen new key kinase targets involved in tumor development could provide new strategies for the developmental process pertaining to small targeted molecule inhibitors. While scientists are working on the next generation of drugs to overcome resistance, they cannot declare their frustration with targeted drugs. As opposed to those mentioned, resistance will promote better inhibitor synthesis. Desirable properties for further development of next-generation compounds include better safety indicators, improved pharmacologic indicators, access to brain metastases or other shelters, and reversal of primary tumors. We expect small molecule targeted compounds to bring good news to cases subjected to advanced carcinoma and open a bright way to solve carcinoma.

## Author Contributions

All authors listed have made a substantial, direct and intellectual contribution to the work, and approved it for publication.

## Conflict of Interest

The authors declare that the research was conducted in the absence of any commercial or financial relationships that could be construed as a potential conflict of interest.

## Publisher’s Note

All claims expressed in this article are solely those of the authors and do not necessarily represent those of their affiliated organizations, or those of the publisher, the editors and the reviewers. Any product that may be evaluated in this article, or claim that may be made by its manufacturer, is not guaranteed or endorsed by the publisher.

## Footnotes

1
https://www.cancer.gov/about-cancer/treatment/drugs
